# Sleep and Activity Patterns in Depression From Wearable Data: Unsupervised Clustering Study

**DOI:** 10.2196/86900

**Published:** 2026-06-10

**Authors:** Carolin Oetzmann, Yuezhou Zhang, Nicholas Cummins, Ewan Carr, Faith Matcham, Sara Siddi, Femke Lamers, Daniel Leightley, Katie M White, Amos A Folarin, Peter Annas, Josep Maria Haro, Brenda WJH Penninx, Srinivasan Vairavan, Til Wykes, Richard JB Dobson, Vaibhav A Narayan, Matthew Hotopf

**Affiliations:** 1 Department of Psychological Medicine, Institute of Psychiatry, Psychology & Neuroscience, King's College London, 16 De Crespigny Park, London, SE5 8AF, United Kingdom, +44 20 7848 0002; 2 Department of Biostatistics & Health Informatics, Institute of Psychiatry, Psychology & Neuroscience, King’s College London, London, United Kingdom; 3 School of Psychology, University of Sussex, Falmer, United Kingdom; 4 Parc Sanitari Sant Joan de Deu, Sant Joan de Deu Research Institute (IRSJD), Sant Boi de Llobregat, Spain; 5 Mental Health Networking Biomedical Research Centre (CIBERSAM), Madrid, Spain; 6 Department of Psychiatry, Amsterdam UMC, Amsterdam, The Netherlands; 7 Amsterdam Public Health, Mental Health Program, Amsterdam, The Netherlands; 8 School of Population Health Sciences, King’s College London, London, United Kingdom; 9 H. Lundbeck A/S, Copenhagen, Denmark; 10 Janssen Research and Development, LLC, Titusville, NJ, United States; 11 Department of Psychology, Institute of Psychiatry, Psychology and Neuroscience, King's College London, London, United Kingdom; 12 South London and Maudsley NHS Foundation Trust, London, United Kingdom; 13 Davos Alzheimer’s Collaborative, Geneva, Switzerland

**Keywords:** major depressive disorder, digital phenotyping, wearable devices, sleep patterns, physical activity, unsupervised learning, Gaussian mixture models, hidden Markov models, behavioral subtypes, longitudinal data, personalized psychiatry

## Abstract

**Background:**

Efforts to advance our understanding of depression have long been constrained by the disorder’s vast symptom heterogeneity and by the reliance on self-report, which offers only a partial view of phenotypic expression. Digital phenotyping provides an opportunity to address these core challenges by generating real-time, objective data on behavior and physiology, offering new perspectives on understanding depression phenotypes. Yet, prior efforts to identify such objectively derived subtypes have relied on predefined diagnostic labels or supervised models, limiting discovery to existing clinical categories.

**Objective:**

This study aimed to identify subtypes of depression based on objective sleep and activity data using an unsupervised learning method and to explore how participants transition between these subtypes over time.

**Methods:**

We analyzed longitudinal Fitbit data from 623 participants with recurrent depression enrolled in the Remote Assessment of Disease and Relapse in Major Depressive Disorder study. To identify our subtypes, we applied Gaussian mixture models and hidden Markov models, incorporating a thorough model selection approach that combined grouped cross-validation and seed selection to ensure robustness.

**Results:**

Three activity subtypes (high, light, and low activity) and 4 sleep subtypes (efficient early sleepers, efficient late sleepers, disrupted sleepers, and variable late sleepers) were consistently identified. These subtypes align with known associations between depression and behavioral patterns. Transition modeling revealed stability within individuals over follow-up, further suggesting the presence of behavioral phenotypes rather than momentary fluctuations.

**Conclusions:**

The results demonstrate that wearable-derived features can identify reproducible and clinically relevant behavioral subtypes of sleep and activity in individuals with major depressive disorder. These subtypes reflect known behavioral correlates of depression and may offer a data-driven framework for reducing phenotypic heterogeneity, improving research stratification, and supporting personalized patient monitoring. Further work is needed to validate these findings in independent cohorts and evaluate their potential use in reducing noise when using sleep or activity data to predict depression outcomes.

## Introduction

Conventional clinical assessment of depression has predominantly relied on interview-based and self-report measures, which are widely used and clinically informative but may be susceptible to biases and variability in administration and adherence [1,2]. These subjective data can be influenced by factors such as recall bias, social desirability effects, and high interrater variability [3]. With the widespread adoption of smartphones [4] and wearable technologies [5] over the past decade, there is an opportunity to improve the way depression is assessed. Digital phenotyping uses remote measurement technologies (RMTs), such as smartphones or wearable devices, to unobtrusively collect continuous real-time data on patient behavior and physiology, such as heart rate, physical activity, or sleep [2]. These data, collected in a naturalistic setting, provide objective correlates that could help reveal a more comprehensive understanding of individual differences in how subjective depression symptoms manifest in daily life [6].

Sleep problems, circadian rhythm disturbances, and physical inactivity have long been recognized as important and potentially modifiable risk factors for depression [7]. These factors exist within a complex interplay of biological, psychological, and environmental processes and can be influenced through behavioral and clinical interventions. Consistent with their clinical relevance, sleep disturbances or alterations in activity levels are recognized as core features of depression symptomatology [8], weighted equally as emotional and cognitive symptoms in gold-standard diagnostic criteria such as the *Fifth Edition of the Diagnostic and Statistical Manual* (*DSM-5* [9]) and the *Eleventh Revision of the International Classification of Diseases* (*ICD-11* [10]).

The idea of using digital phenotypes to better monitor depression states and symptoms is not new; previous research has established an association between RMT features and depression severity or clinical state [11-13]. For example, earlier studies have consistently identified individuals with depression as having lower physical activity levels, quantified as reduced overall movement, less vigorous activity, or higher sedentary behaviors, compared to controls [14-16]. These associations suggest a general link between lower physical activity and depression and indicate heterogeneity in the ways patients might exhibit reduced activity. This variability, consistent with the broader heterogeneity of depression presentation [17], may point to latent subgroups defined by patterns of physical activity, although this remains speculative.

A large body of literature on sleep identifies diverse dysregulation profiles in sleep patterns and circadian rhythms as both markers of, and risk factors for, depression [11,18,19]. Some studies show that clinical groups exhibit higher nighttime activity than controls [20], while others link evening chronotypes [21] and increased sleep variability [22] to depression. These sleep disturbances indicate that depression manifests differently across individuals, potentially revealing distinct patient subtypes defined by specific sleep patterns. Current diagnostic criteria for major depressive disorder (MDD) include both insomnia and hypersomnia. But the significant co-occurrence rates (up to 30%) suggest that sleep is more complex than these 2 dimensions imply [23].

By using unsupervised clustering techniques [24] on sleep and activity data, we can investigate potential subtypes or patterns that may be revealed in the RMT data alone. This approach contrasts with prior work, which has largely relied on predetermined labels or subjective patient information. In doing so, it enables the identification of potential latent subgroups within a phenotypically diverse sample of individuals with depression. This paper addresses this gap in the literature by (1) identifying subtypes of depression from objective sleep and activity data using an unsupervised learning method and (2) exploring how participants transition between these subtypes over time.

## Methods

### Study Design and Participants

This study was a secondary analysis of the Remote Assessment of Disease and Relapse Study in Major Depressive Disorder (RADAR-MDD) [25] conducted to explore subtypes of depression using objective sleep and activity data collected via a Fitbit wearable device. The RADAR-MDD study was a longitudinal observational cohort study involving patients with a history of recurrent MDD and aimed to monitor the illness course using remote measurement technologies, such as a Fitbit wearable device.

The study enrolled 623 participants who (1) fulfilled *DSM-5* diagnostic criteria for MDD within the past 2 years and (2) had experienced at least 2 depressive episodes in their lifetime. The exclusion criteria included a history of bipolar disorder, dementia, psychosis or MDD with psychotic features, a history of moderate-to-severe drug or alcohol dependence in the last 6 months, or a history of major medical disease that might impact their ability to participate in normal daily life [25]. Participants were enrolled and followed up for an average of 18 months (range 11‐36 months). They were asked to wear the Fitbit device throughout the study and complete a battery of questionnaires every 3 months.

The study ran from November 30, 2017, to April 30, 2021, across 3 sites: King’s College London (UK), Amsterdam University Medical Centre (the Netherlands), and Centro de Investigación Biomédica en Red (Spain). Participants were recruited from diverse sources, including volunteer registers of people with MDD and clinical samples of people attending mental health care services. The full RADAR-MDD protocol [25] and data availability, cohort profile, and retention [26] have been published elsewhere.

### Ethical Considerations

The study obtained ethical approval from the Camberwell St Giles Research Ethics Committee (REC reference: 17/LO/1154) in London, from the CEIC Fundacio Sant Joan de Deu (CI: PIC-128‐17) in Barcelona, and from the Medische Ethische Toetsingscommissie VUmc (METc VUmc registratienummer: 2018.012—NL63557.029.17—registration date: September 10, 2018) in the Netherlands. All participants provided written informed consent.

### Lived Experience Involvement

The RADAR-MDD lived experience group was instrumental in co-designing the study and data collection processes, including the types and frequencies of data collection, as well as contributing to problem-solving throughout the study, as detailed further in Simblett et al [27].

### Measures

#### Three Monthly Outcome Assessments

Throughout the follow-up period, every 3 months, participants were asked to complete a battery of questionnaires; these included, among others, the Inventory of Depressive Symptomatology–Self Report (IDS-SR; [28]), the questionnaire assessing the 7-item Generalized Anxiety Disorder (GAD-7; [29]), and the Work and Social Adjustment Scale (WSAS; [30]).

#### Digital Sleep and Activity Data

Digital sleep and activity data were collected through Fitbit Charge 2 and later the Charge 3 wearable devices, which recorded information on sleep and physical activity throughout the follow-up period. Table 1 summarizes the sleep and activity features included in the analysis. Of the features available for extraction, these were deemed to provide the most comprehensive and clinically relevant picture of aggregated sleep and activity. Previous literature reviews have identified them as some of the most frequently included digital features in studies linking depression and RMTs [11,13,14,31,32].

**Table 1.** Digital feature definitions for the variables included in the subtyping solutions.

Domain and digital feature	Definition
Sleep features	
Total sleep time	Total duration (sum) of all “nonawake” stages
Sleep efficiency	Percentage of total sleep time to time in bed
Time awake in bed	Percentage of total time in bed spent awake
Sleep onset time	Onset time recorded in local time as a decimal number of hours (eg, 22.5=10:30 PM)
Sleep offset time	Offset time recorded in local time as a decimal number of hours (eg, 6.65=6:39 AM)
Activity features	
Sedentary time	Number of minutes labeled “sedentary” all day
Light activity	Number of minutes labeled “lightly active” all day
Moderate activity	Number of minutes labeled “fairly active” all day
Vigorous activity	Number of minutes labeled “very active” all day
Nighttime activity	Number of “active” minutes during nighttime (00:00-05:59)
Total daily calories	Sum of calories per minute along all day (ie, calories per day)

#### Baseline Sociodemographic and Clinical Characteristics

Assessed at baseline, sociodemographic and clinical characteristics were used to describe differences in the resulting solutions (see Table 2 for the variables included). These variables were chosen based on data availability and clinical relevance.

**Table 2.** Sociodemographic and clinical characteristics used to describe subtype membership^a^.

Variable	Further information
Age	Age at enrollment
Gender	Female, male, prefer not to say
Employment status	Employed or furloughed Retired Student Unemployed or sick leave
Family history of depression	Having a parent, sibling, and/or child with a diagnosis of depression
Mental health comorbidity^a^	Presence of a mental health comorbidity
Physical health comorbidity^a^	Presence of a physical health comorbidity
Depression*	The Inventory of Depressive Symptomatology–Self Report (IDS-SR; [28])
Anxiety*	Questionnaire assessing 7-item Generalized Anxiety Disorder (GAD-7; [29])
Functional impairment*	Work and Social Adjustment Scale [30]

a Mental and physical health comorbidity groups are not mutually exclusive. All variables were assessed at baseline, except for those marked with an asterisk (*), which were also assessed during the 3-month outcome assessments.

### Data Processing, Missing Data Handling, and Outliers

Each completed 3-monthly questionnaire assessment was paired with digital data collected up to 7 days before questionnaire completion. Among the measures, the IDS-SR was selected as the primary criterion for determining whether a 3-monthly assessment was included in the analysis due to its 80% completion rate [26] and significant clinical relevance in assessing depression symptoms. Since the IDS-SR asks respondents to reflect on their symptoms over the past week, a 7-day window was chosen for pairing it with digital data.

Aligning the RMT measures with this time frame was imperative (1) to guarantee that the self-reported symptom experiences participants reflect upon for the IDS-SR would mirror the digitally collected data sources, (2) to ensure consistency in data processing and cleaning across publications (eg, Oetzmann et al [33]), and (3) to allow for future prediction models where the IDS-SR is the outcome measure.

Sleep and activity data were summarized into weekly means and SDs. A missing data threshold of at least 12 hours per day of the available data was used to ensure thorough, reliable summaries of digital features that account for a broad range of daily or nightly behavior changes [34]. Additionally, to provide a realistic view of each week, participants with fewer than 3 days of data within the 7 days before IDS-SR completion were excluded, informed by previous work [35]. The number of observations between a 3-day and 4-day cut-off was minimal (1748 vs 1697 in the activity sample). Given this difference, the more inclusive 3-day cut-off was used to retain a larger dataset. Due to inconsistencies in the raw data during preprocessing, the variable “time awake in bed (SD)” could not be reliably extracted and was therefore excluded from the final model.

This cleaning was undertaken in the activity and sleep data separately to help reduce data loss. As a result, the final datasets for both sleep and activity have varied levels of completeness, with different numbers of participants and observations in each. This variation is accounted for and reported in the results for each corresponding modeling solution. Any remaining missingness within the 2 datasets was assessed and imputed using k-nearest neighbors (*k*=5) before clustering [36]. Missingness was minimal, and imputation was applied to preserve observations and maintain complete longitudinal trajectories.

### Clustering Analysis

#### Overview

We decided to analyze sleep and activity data separately because (1) despite their inherent connection, they represent distinct behavioral and physiological constructs, (2) the exploratory nature of this analysis benefits from focusing on each domain independently, avoiding interference or dilution of signals, and (3) it simplifies model complexity and enhances interpretability.

Following best practice guidelines [24], sleep and activity data were explored using 2 probabilistic unsupervised machine learning–based clustering approaches: Gaussian mixture models (GMMs; [37]), a static model, and hidden Markov models (HMMs) with Gaussian emissions [38], which consider repeated measures. Using 2 complementary approaches, we could check whether the solution was highly dependent on the algorithm used or method-agnostic, suggesting better generalizability.

Clustering was conducted at the observational level, with baseline and follow-up timepoints included simultaneously in each model. Thus, participants contributing multiple timepoints could be assigned to different clusters across assessments. In the GMM approaches, observations were treated as independent, consistent with identifying cross-sectional latent structure across all observations. In contrast, the HMM explicitly incorporated the temporal ordering of repeated measures, enabling the estimation of transition probabilities that describe movement between states over time.

#### Model Specifications and Software

We used a diagonal covariance matrix for the GMM and the HMM, as it requires less computational power than a full matrix. Additionally, it reduces the risk of overfitting in smaller datasets (compared to a full matrix) [39]. For the GMM, we used “k-means++” as the setting for the initialization parameter to ensure that the initial cluster centers are spread out in the data space, thereby avoiding convergence on a suboptimal solution. This also reduces computational cost compared to running a full k-means [39]. All other model specifications were set to default according to the Gaussian HMM model in the hmmlearn package [40] and the GaussianMixture in the Scikit-learn toolkit [39,41]. All analyses were conducted using Python (version 3.11.0) [42]. All figures were created in R (version 4.3.1) [43] using the ggplot2 package [44].

#### Selection of the Optimal Number of Clusters or State Solutions

**Figure 1.**
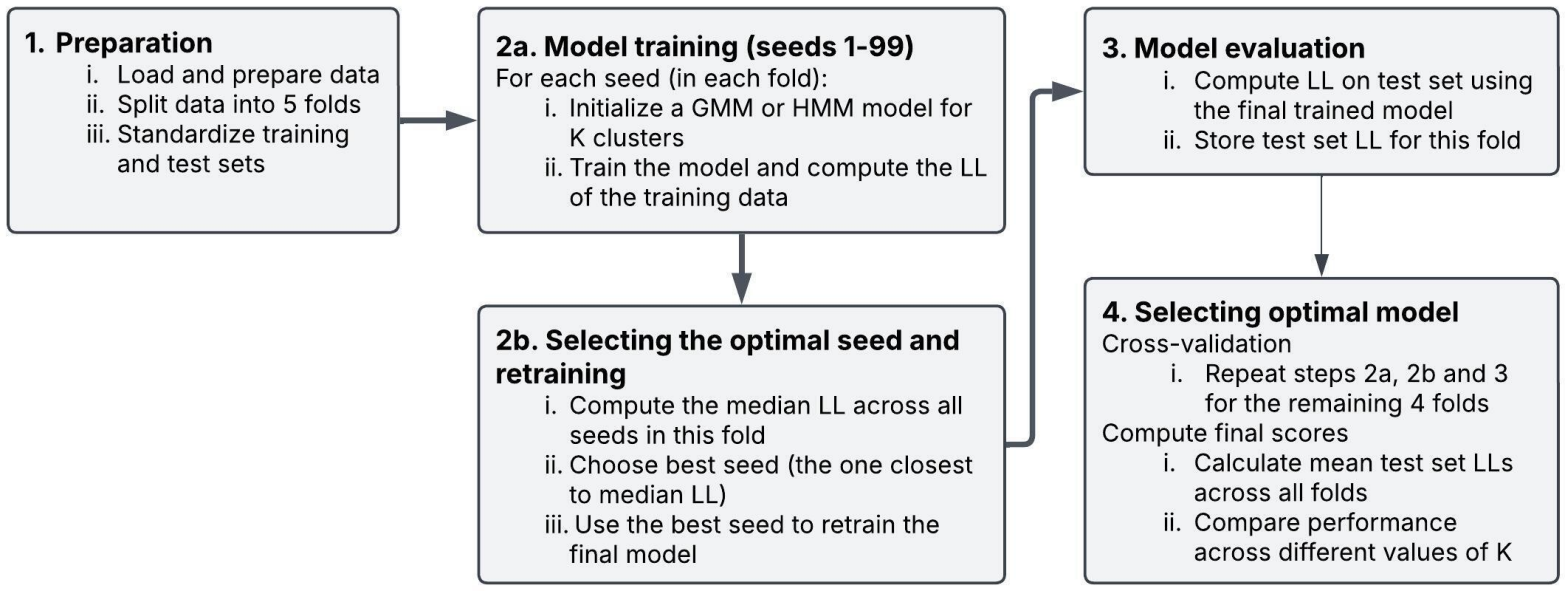
Step-by-step process for Gaussian mixture models (GMM) or hidden Markov models (HMM) clustering and model selection.

We undertook several steps to determine the optimal number of clusters or states for the GMM and the HMM separately. Our approach aimed to ensure that the chosen model complexity was based on robust, generalizable performance rather than overfitting or sensitivity to random initializations. For each algorithm, we tested 2‐10 cluster or state solutions, with 5 folds stratified by participant identifier, to ensure that all observations for a particular participant were always in the same set. The process is outlined in Figure 1.

1. Data preparation. First, the data were split into 5 folds grouped by participant using GroupKFold. In each cross-validation iteration, 4 folds were used for training, and the remaining fold served as the test set; no separate hold-out dataset was created. The features in the training data were standardized, which involved centering the data by subtracting the mean and scaling it to have an SD of 1 (mean 0, SD 1). This same transformation was then applied to the testing dataset to prevent data leakage.
2. Model training and seed selection. To reduce sensitivity to random initialization, each model (per fold and cluster number) was trained across 99 seeds, and the seed with the log-likelihood (LL) closest to the median was selected to initialize the final “median-fit” model. LL was used because it reflects model fit under the assumed probabilistic framework, providing an objective and comparable measure across initializations. This approach, analogous to burn-in or stabilization steps used in other clustering algorithms, provides a robust and generalizable solution. The median model seed was chosen because the LL is sensitive to initialization [45]; selecting the median corresponds to the middle ground initialization that is robust to this variability, ensuring a stable and generalizable solution.
3. Evaluating the “median-fit” model on test data. Next, we estimated the test set log-likelihood score by evaluating the test data on the “median-fit” model, providing a test LL score that indicates how well the model generalizes to unseen data. Each of these 5 resulting test LL scores (1 per fold) for K number of clusters is then stored for subsequent analysis.
4. Selecting the optimal number of clusters. Once these steps are repeated for (2‐10 cluster solutions), the mean of the test LL scores for each fold for each cluster solution is then calculated and plotted on a graph. The optimal number of clusters is then selected by identifying the point at which the curve hits a plateau, indicating that the gain in fit from adding additional clusters is no longer substantial.

#### Model Fitting and Cluster Interpretation

Once the optimal number of clusters was identified using grouped 5-fold cross-validation, the model was refitted on the full dataset (ie, all 5 folds combined) using the selected number of clusters and the corresponding “median-fit” seed. No separate test set was retained at this stage because generalization performance had already been estimated via cross-validation. This model was then refitted, and cluster assignments (state labels) were predicted for each observation (ie, each observation was assigned to its most likely cluster). Once each observation was assigned to a given cluster, descriptive statistics for each profile were calculated. For each cluster, the mean values of the input RMT metrics were computed and interpreted to ascertain digital feature differences between the identified clusters. To facilitate comparisons across different features, we used the standardized and centered feature values, representing standardized deviations from the population mean for both the GMM and HMM solutions. For ease of interpretation, Tables S1 and S2 in Multimedia Appendix 1 present the HMM solution in raw units for the sleep and physical activity clusters, respectively. This was only done for the HMM, as the GMM and HMM solutions showed high comparability.

#### Demographic and Clinical Cluster Differences

Finally, the demographic and clinical characteristics of the states were identified. Descriptive statistics for baseline and follow-up variables were calculated at the observation level, meaning that participants contributing multiple follow-ups appeared multiple times. No formal statistical tests were performed due to the nonindependence of repeated observations. These analyses were exclusively reported on the HMM solution, as the HMM considers repeated measures, and the obtained solutions showed high comparability with those derived from the GMM model.

#### Transition Probability Estimation

For the HMM, transition probabilities between states were estimated as part of the model fitting. These probabilities represent the probability of transitioning from one state at a given observation to another state at the subsequent observation within the same participant. Transition estimates were derived from all available consecutive 3-month time points per participant, allowing the model to characterize within-person movement between behavioral states over time. Participants contributed transitions only when sequential observations were available.

## Results

### Sample Characteristics

The overall sample of the RADAR-MDD study consisted of 623 participants; further details on the entire cohort’s sample characteristics are reported in Matcham et al [26]. This paper used an analytical sample of 490 participants (2030 observations) for the sleep clustering work and 472 participants (1748 observations) for the activity clustering. Table 3 presents the demographic and clinical characteristics of the overall sample and 2 analytical samples, showing no substantial differences across variables, indicating their comparability.

**Table 3.** Sample characteristics for the total sample, sleep, and activity analytical samples.

Characteristics	RADAR-MDD^a^ sample (n=623; obvs.=3724)	Sample 1	Sample 2
		Sleep data (n=490; obvs.=2030)	Activity data (n=472; obvs.=1748)
Study site, n (%)			
United Kingdom	350 (56.2)	276 (56.3)	271 (57.4)
Spain	155 (24.9)	107 (21.8)	115 (24.4)
The Netherlands	118 (18.9)	107 (21.8)	86 (18.2)
Age, mean (SD)	46.4 (15.3)	45.6 (15.3)	46.3 (15.2)
Gender, n (%)			
Female	471 (75.6)	371 (75.7)	351 (74.4)
Male	152 (24.4)	119 (24.3)	121 (25.6)
Aggregated ethnicity, n (%)			
White British or Dutch	369 (59.2)	314 (64.1)	291 (61.7)
White other	35 (5.6)	28 (5.7)	25 (5.3)
Black ethnic group	14 (2.2)	9 (1.8)	11 (2.3)
Asian ethnic group	16 (2.6)	9 (1.8)	11 (2.3)
Mixed ethnic group	16 (2.6)	10 (2)	9 (1.9)
Other	18 (2.9)	13 (2.7)	10 (2.1)
Not reported^b^	155 (24.9)	107 (21.8)	115 (24.4)
Baseline depression score (IDS-SR)^c^			
IDS-SR total, mean (SD)	31.3 (14.5)	30.8 (14.4)	31.2 (14.3)
None (0‐13), n (%)	61 (9.8)	50 (10.2)	44 (9.3)
Mild (14-25), n (%)	157 (25.2)	131 (26.7)	123 (26.1)
Moderate (26-38), n (%)	206 (33.1)	164 (33.5)	158 (33.5)
Severe (39-48), n (%)	104 (16.7)	74 (15.1)	80 (16.9)
Very severe (49-84), n (%)	79 (12.7)	61 (12.4)	56 (11.9)
Not reported*,* n (%)	16 (2.6)	10 (2.0)^d^	11 (2.3)^d^

a RADAR-MDD: Remote Assessment of Disease and Relapse in Major Depressive Disorder.

b Ethnicity data were not collected at the Spanish site (n=155).

c IDS-SR: Inventory of Depressive Symptomatology–Self Report.

d These individuals missed baseline IDS-SR measures but provided follow-up IDS-SR measures, which were included in the analysis.

### Missing Data

After data cleaning and preprocessing, the resulting data for sleep and activity had marginal amounts of missingness, with the sleep data showing 7 missing values out of 2030 for the IDS-SR total score. In the activity dataset, 8 out of 1748 missing values were found for nighttime activity (mean) and 23 out of 1748 for nighttime activity (SD). This missingness was deemed small and imputed using K-nearest neighbors’ imputation.

### Digital Sleep Data Clustering

#### Selecting the Optimal Number of Clusters

Figure S1 in Multimedia Appendix 1 presents the mean LL scores across the 5 folds evaluated on the unseen test data for a given number of clusters. In the GMM solution, LL increased substantially up to 4 clusters, with more modest improvements thereafter. Although the curve continued to rise slightly beyond 4 clusters, the 4-cluster solution was selected to balance model fit, parsimony, and interpretability. Similarly, the HMM solution showed substantial improvement up to 4 states, followed by progressively smaller gains with additional states, indicating diminishing returns beyond this point. Taken together, these patterns support the selection of the 4-cluster solution across both the GMM and HMM solutions.

#### Description

**Figure 2.**
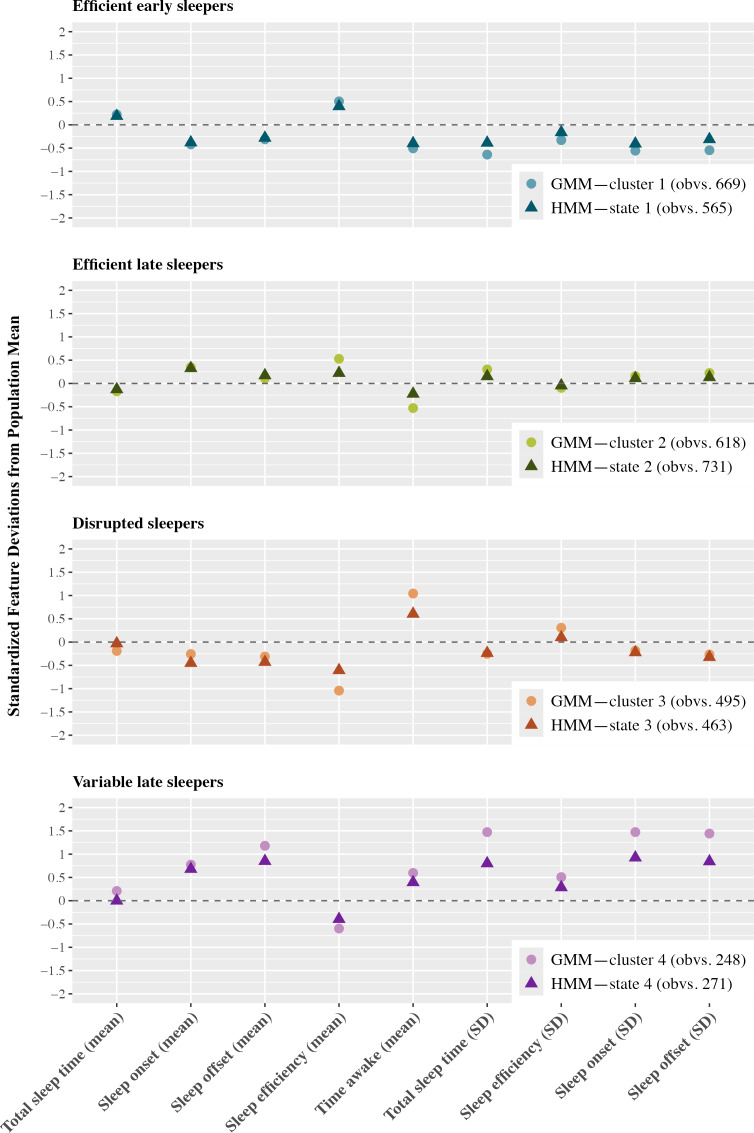
Descriptive means for the sleep features included in the 4-cluster Gaussian mixture model (GMM) and 4-state hidden Markov model (HMM) solutions. Obvs.: number of observations in the state or cluster. The data values are presented in Table S3 in Multimedia Appendix 1.

The 4-cluster GMM and 4-state HMM solutions have similar descriptive characteristics across the digital sleep features used to define the model. The 4-cluster solution for the GMM and the 4-state solution for the HMM are visualized in Figure 2, presenting deviations from the population mean. Table S1 in Multimedia Appendix 1 presents the findings in raw units without scaling for the HMM solution. Overall, these results indicate the following:

- State 1 or cluster 1 (ie, efficient early sleepers) had higher-than-average sleep efficiency and lower-than-average awakenings (an average sleep efficiency of 92.4% and 7.6% of the time in bed spent awake), with earlier-than-average sleep onset and offset times (mean) (an average onset at 23:40 and offset at 7:39) and lower-than-average levels of sleep onset and sleep offset time (SD) variation, indicating consistent sleep patterns night-to-night.
- State 2 or cluster 2 (ie, efficient late sleepers) had higher-than-average sleep efficiency (average sleep efficiency of 92% and 8% of time spent awake) and lower-than-average awakenings. They had later-than-average sleep onset and offset times (mean), with a mean sleep onset at 00:47 (the next day) and offset at 8:25.
- State 3 or cluster 3 (ie, disrupted sleepers) had lower-than-average sleep efficiency levels and higher-than-average levels of awakenings (mean sleep efficiency of 90.2% and 9.8% of the time spent awake). They presented earlier-than-average sleep onset and offset time (mean) compared to the overall population (an average onset time of 23:32 and an offset at 07:24).
- State 4 or cluster 4 (ie, variable late sleepers) had the highest levels of variation (around 2 hours each) in total sleep time (SD), sleep onset, and offset time (SD) over the 7 days compared to the population’s mean variation. They presented with lower-than-average efficient sleep (90.6%), spending approximately 9.4% of their time in bed awake, and later-than-average sleep onset and offset times (01:22 the next day and 09:33, respectively).

#### Demographic and Clinical Characteristics of the Sleep States

Table 4 describes the demographic and clinical characteristics of each state. The clusters presented similar characteristics, with only slight differences observed across variables. For state 1, efficient early sleepers had a slightly higher proportion of female participants than the overall sample. In contrast, in state 4, variable late sleepers had the highest proportion of male participants. When considering employment, state 4, variable late sleepers, had a slightly lower proportion of people employed or furloughed and a higher proportion of unemployed or sick leave individuals compared to the overall. The prevalence of physical health comorbidities varied across states; state 1 (efficient early sleepers) had the least, while state 4 (variable late sleepers) had the highest presence of physical comorbidities. No substantial differences in depression severity and anxiety were observed; all states fell within the moderate range on the IDS-SR, though a 10-point difference was observed between state 1 (efficient early sleepers; 27.1) and state 4 (variable late sleepers; 38.4), highlighting potentially clinically relevant variation within the same severity category. States 2 (efficient late sleepers) and 4 (variable late sleepers) exhibited marginally higher GAD-7 anxiety scores compared to the other states and were slightly more affected by functional disability, indicating significant impairments.

**Table 4.** Demographic and clinical characteristics of each state identified in the hidden Markov model (HMM) sleep feature solution.

		State 1	State 2	State 3	State 4
Variable	Overall	Efficient early sleepers	Efficient late sleepers	Disrupted sleeper	Variable late sleepers
Observations, n (%)	2030 (100)	565 (27.8)	731 (36)	463 (22.8)	271 (13.3)
Age (y), mean (SD)	47.4 (14.9)	47 (15)	45 (14.5)	51 (14.3)	48 (15.4)
Gender, n (%)					
Female	1577 (77.7)	488 (86.4)	561 (76.7)	357 (77.1)	171 (63.1)
Study site, n (%)					
United Kingdom	1231 (60.6)	354 (62.7)	434 (59.4)	297 (64.1)	146 (53.9)
Spain	415 (20.4)	86 (15.2)	172 (23.5)	83 (17.9)	74 (27.3)
The Netherlands	384 (18.9)	125 (22.1)	125 (17.1)	83 (17.9)	51 (18.8)
Employment status, n (%)					
Employed or furloughed	898 (44.2)	273 (48.3)	336 (46)	201 (43.4)	88 (32.5)
Retired	444 (21.9)	130 (23)	125 (17.1)	118 (25.5)	71 (26.2)
Student	193 (9.5)	52 (9.2)	79 (10.8)	37 (8)	25 (9.2)
Unemployed or sick leave	383 (18.9)	96 (17)	141 (19.3)	85 (18.4)	61 (22.5)
Other or not available	112 (5.5)	14 (2.5)	50 (6.8)	22 (4.8)	26 (9.6)
Family history of depression, n (%)					
Yes	1470 (72.4)	411 (72.7)	511 (69.9)	355 (76.7)	193 (71.2)
Mental health comorbidity, n (%)^a^					
Yes	1398 (68.9)	386 (68.3)	542 (74.1)	293 (63.3)	177 (65.3)
Physical health comorbidity, n (%)^a^					
Yes	1017 (50.1)	219 (38.8)	398 (54.4)	211 (45.6)	189 (69.7)
IDS-SR^b^, mean (SD)	31.0 (15.3)	27.1 (14.7)	32.8 (15)	28.5 (14)	38.4 (16)
WSAS^c^, mean (SD)^d^	18.8 (11.1)	15.9 (11)	20.3 (10.5)	17.7 (11.1)	22.2 (11)
GAD-7^e^, mean (SD)^d^	8.3 (5.4)	7.5 (5.3)	9.1 (5.2)	7.6 (5.2)	9.1 (6)

a Mental health and physical health comorbidity are not mutually exclusive groups.

b IDS-SR: Inventory of Depressive Symptomatology–Self Report. Scores of 26-38 indicate moderate levels of depression, and scores of 39-48 indicate severe levels of depression [28].

c WSAS: Work and Social Adjustment Scale. Scores of 11-20 indicate some impairment, and scores >20 indicate significant impairment [30].

d Indicates variables with missing data. The WSAS had 30 missing entries, and the GAD-7 had 14 entries.

e GAD-7: 7-item Generalized Anxiety Disorder. Scores ≥10 indicate moderate-to-severe anxiety [29].

### Digital Physical Activity Data Clustering

#### Selecting the Optimal Number of Clusters

Figure S2 in Multimedia Appendix 1 presents the mean LL scores across the 5 folds evaluated on the unseen test data, for the physical activity models. For the GMM, a clear inflection point is observed at 3 clusters. This suggests that a 3-cluster solution provides the best fit for the data. The HMM LL curve shows an inflection point at 3 and 7 states. Based on these findings, the 3-state GMM and HMM solutions were selected as the optimal number of clusters. The 7-state HMM was also explored and is presented in Table S4 in Multimedia Appendix 1 for further reference.

#### Description

**Figure 3.**
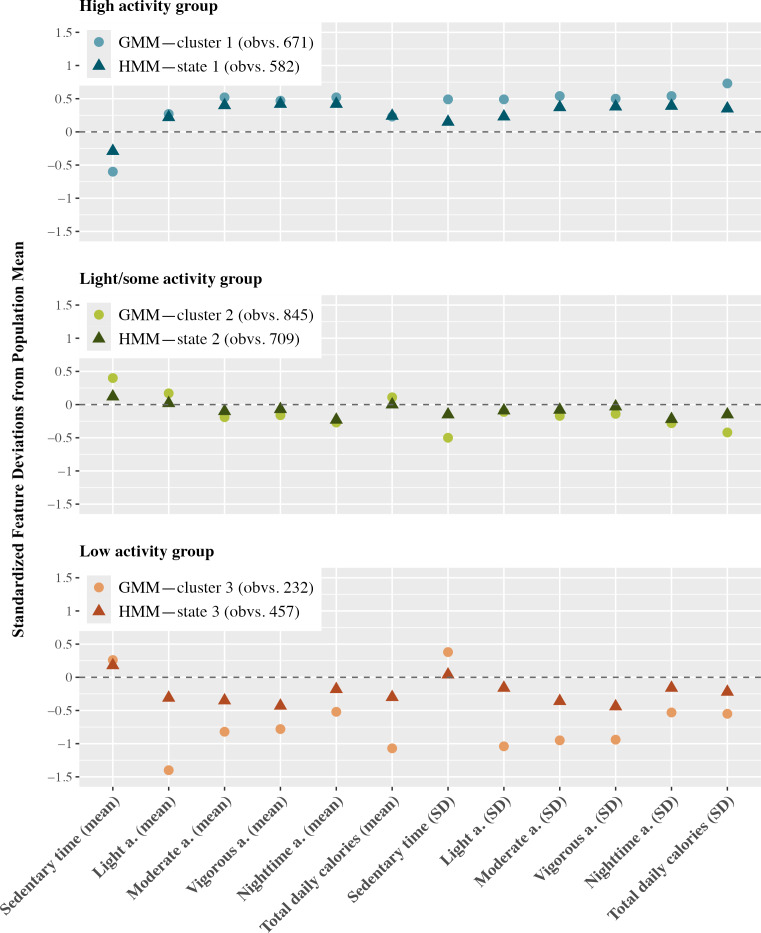
Descriptive means for the physical activity features included in the 3-cluster Gaussian mixture model (GMM) and 3-state hidden Markov model (HMM) solutions. A.: activity; Obvs.: number of observations in the state or cluster. The data values are presented in Table S5 in Multimedia Appendix 1.

The 3-cluster solutions for the GMM and HMM models presented similar solutions. Figure 3 describes these differences, presenting deviations from the population mean. Table S2 in Multimedia Appendix 1 presents the findings in raw units without scaling for the HMM solution.

- State 1 or cluster 1 (ie, high activity) had the lowest mean levels of sedentary time compared to any other cluster (18.5 h), the highest levels of light, moderate, and vigorous activity (around 3 h, 26 min, and 26 min, respectively), and the highest level of variation in activity levels compared to the population mean (around 20 minutes for moderate and vigorous activity).
- State 2 or cluster 2 (ie, light or some activity) presented with above-average levels of sedentary time and slightly above-average levels of light activity compared to the population mean. This translates to around 19.5 hours of sedentary time, around 3 hours of light activity, 15 minutes of moderate, and 15 minutes of vigorous activity. All measures of variation in activity showed results near the total sample mean.
- State 3 or cluster 3 (ie, low activity) presented the lowest levels of light, moderate, and vigorous activity compared to any other cluster and slightly above-average sedentary time. This translates to a mean of 2.5 hours in light activity, 10 minutes in moderate, and 8 minutes in vigorous activity but a sedentary time of nearly 20 hours.

#### Demographic and Clinical Characteristics of the Activity States

Their demographic and clinical characteristics were explored to examine the distinctions among the 3 delineated activity groups. The states varied only marginally across characteristics. Table 5 presents these differences, showing state 1 (high activity) to have the highest proportion of male participants (n=198, 34%) and instances of no physical health comorbidity (n= 335, 57.6%) compared to any other cluster or the overall sample. State 3 (low activity) had a slightly higher proportion of retired participants than the overall sample (n= 126, 27.6% and n= 383, 21.9%, respectively). Clinical differences are minor, with all states presenting moderate levels of depression (scores between 26 and 38). State 3 (low activity) presented the highest moderate IDS-SR score compared to the other 2 groups. All states presented some functional impairment (scores between 11 and 20) and mild anxiety scores between 5 and 9.

**Table 5.** Demographic and clinical characteristics of each state identified in the hidden Markov models (HMM) physical activity feature solution.

		State 1	State 2	State 3
Variable	Overall	High activity group	Light or some activity	Low activity
Observations, n (%)	1748	582 (33.3)	709 (40.6)	457 (26.1)
Age, mean (SD)	47.3 (14.6)	46.6 (14.8)	46.8 (14.5)	48.8 (14.4)
Gender, n (%)				
Female	1297 (74.2)	384 (66)	554 (78.1)	359 (78.6)
Study site, n (%)				
United Kingdom	1153 (66)	334 (57.4)	535 (75.5)	284 (62.1)
Spain	393 (22.5)	141 (24.2)	124 (17.5)	128 (28)
The Netherlands	202 (11.6)	107 (18.4)	50 (7.1)	45 (9.8)
Employment status, n (%)				
Employed or furloughed	787 (45.0)	275 (47.3)	329 (46.4)	183 (40)
Retired	383 (21.9)	116 (19.9)	141 (19.9)	126 (27.6)
Student	158 (9)	44 (7.6)	77 (10.9)	37 (8.1)
Unemployed or sick leave	332 (19)	123 (21.1)	133 (18.8)	76 (16.6)
Other or Not available	88 (5)	24 (4.1)	29 (4.1)	35 (7.7)
Family history of depression, n (%)				
Yes	1276 (73)	416 (71.5)	530 (74.8)	330 (72.2)
Mental health comorbidity, n (%)^a^				
Yes	1198 (68.5)	368 (63.2)	479 (67.6)	351 (76.8)
Physical health comorbidity, n (%)^a^				
Yes	919 (52.6)	247 (42.4)	400 (56.4)	272 (59.5)
IDS-SR^b^, mean (SD)	31.3 (15.4)	28.9 (15.3)	30.9 (15.1)	34.8 (15.2)
WSAS^c^, mean (SD)^d^	18.8 (11.3)	18.5 (11.2)	17.9 (11.3)	20.6 (11.3)
GAD-7^e^, mean (SD)^d^	8.5 (5.5)	8.1 (5.4)	8.2 (5.3)	9.2 (5.6)

a Mental health and physical health comorbidity are not mutually exclusive groups.

b IDS-SR scores of 26‐38 indicate moderate levels of depression, while scores of 39‐48 indicate severe levels of depression [28].

c WSAS: Work and Social Adjustment Scale. Scores of 11‐20 indicate some impairment, and scores >20 indicate significant impairment [30].

d Indicates variables with missing data: WSAS had 30 missing entries, and GAD-7 had 14 missing entries.

e GAD-7: 7-item Generalized Anxiety Disorder. Scores ≥10 indicate moderate-to-severe anxiety [29].

### Transition Probabilities

**Figure 4.**
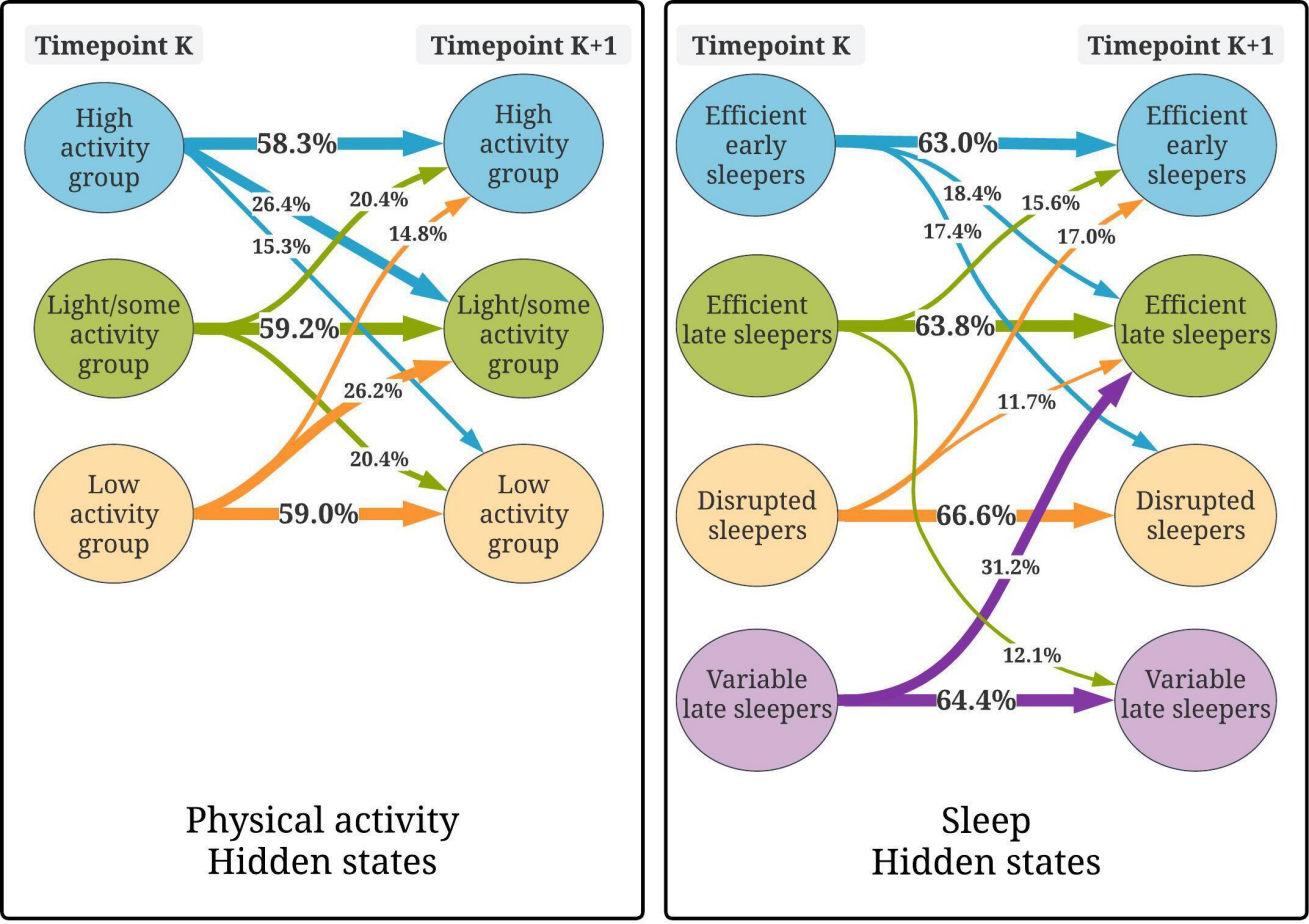
Flowchart indicating transition probabilities as observed in the physical activity hidden Markov models (HMM) and the sleep HMM. Transitions with less than 10% transition probability were omitted to improve readability. Arrows are weighted to highlight more probable transitions.

The likelihood of transitioning between the hidden states according to the sleep and activity HMM models can be visualized in Figure 4. Across both the sleep and the physical activity solutions, the largest observed probabilities were self-transitions, meaning individuals were most likely to remain in the same state. When considering physical activity hidden states specifically, the most likely transitions across different states were from the high activity group to the light or some activity group and from the low activity group to the light or some activity group, with both reporting around a 26% chance of transitioning. Transitions between low- and high-activity groups were less likely, reporting around a 15% chance. When considering sleep, the most likely transition between different states was moving from the variable late sleepers to efficient late sleepers, with a 31.2% chance.

## Discussion

### Principal Findings

This study identified subtypes based on objective sleep and activity data in a heterogeneous sample of participants diagnosed with MDD. It also explored transitions between these subtypes over time. Previous research has shown that various digital features related to sleep and activity can predict the clinical state of depression [11,13]. Based on this, we hypothesized that these same digital markers might be able to uncover nuanced behavioral differences among patients with MDD and identify latent subtypes to provide refined insight into the diverse phenotypes of depression.

**Figure 5.**
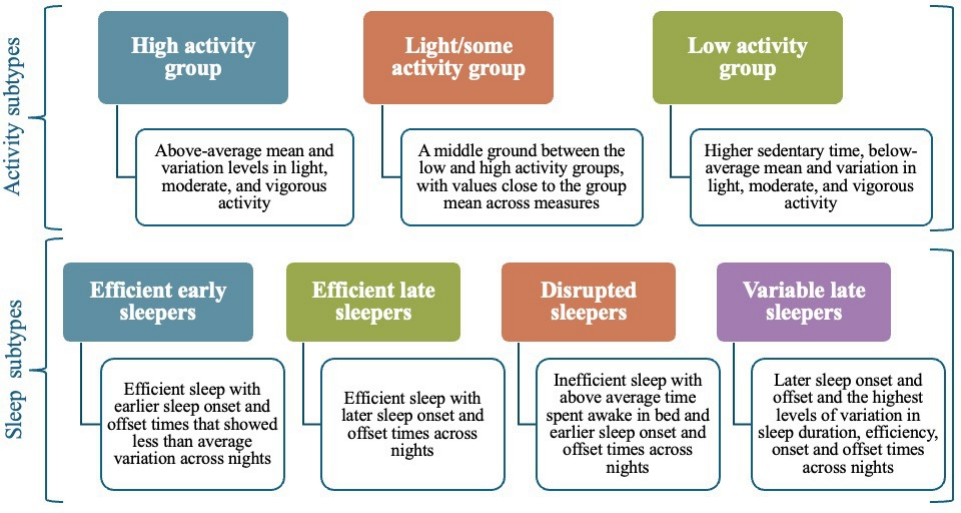
Diagram describing the subtypes identified in the digital sleep and digital physical activity hidden Markov models (HMM) solutions.

To accomplish this, we used cross-fold validation and a seed optimization procedure to derive a stable and reproducible optimal number of clusters in the RADAR-MDD sample. We identified a stable 4-group model as the best fit for sleep feature data and a 3-group solution as the best fit for the physical activity data. The resulting profiles are depicted in Figure 5. Participants were most likely to remain in the same state over time.

### Implications and Comparisons with Previous Work

#### Sleep Profiles Identified via RMT

The human sleep-wake cycle is governed by the interplay between sleep homeostasis and circadian processes. Dysregulation of this system, for example, when an individual’s internal circadian rhythm and external behavior do not align, can cause sleep disruptions [46]. With approximately 90% of patients affected, sleep disturbance is one of the most commonly reported symptoms of depression [47,48]. This study aimed to examine these differences via clustering of sleep RMT data, identifying 4 subtypes in the RADAR-MDD sample.

Notably, variable late sleepers were characterized by later mean sleep-wake patterns, higher awakenings, and inconsistent sleep onset, offset, and durations over the 7 days compared to the overall population. They typically went to bed around 1:22 AM (the following day) and woke around 9:30 AM, with over 2 hours of variability in these times each week. Among others, the observed variability in sleep patterns may indicate social jet lag, where differing weekday and weekend schedules disrupt sleep cycles [49]. This variability to meet social and work demands can negatively impact circadian rhythms, increasing the risk of poor mental and physical health [49,50].

Previous literature looking at sleep profiles in depression frequently draws a distinction between morning and evening preference types [51,52]. Seo et al [53] describe this as morning-types preferring earlier bedtimes and wake-up times, while evening-types prefer later bedtimes and wake-up times according to self-report measures. While we cannot infer personal preference due to the passive nature of the Fitbit sleep data collection, we identify distinct differences in sleep onset and offset times, which mirror similar patterns.

The efficient late sleepers presented similar later sleep-wake patterns (as seen in variable late sleepers) but efficient sleep with little time awake compared to the overall sample, with an average sleep onset of 00:47 (the next day) and offset at 8:25. Previous works examining later sleep-wake patterns using wearables have found associations with worse outcomes [22,54-56]. Studies using self-report metrics have found that a morning preference may be protective against depression symptoms [57], while evening preference is associated with worse adverse effects [18,58]. Previous literature indicates rumination as a key psychological mediator in the relationship between depression and later sleep onset [59].

In contrast, both disturbed sleepers and efficient early sleepers showed earlier than average sleep onset and offset times, with average variability in these timings. They typically began sleeping around 23:30 and woke around 7:30, around 1 to 2 hours earlier compared to their late sleep counterparts and experienced less than an hour of variation in both onset and offset times each week. They also differ, with efficient early sleepers presenting with high sleep efficiency with little time awake in bed, while disturbed sleepers present with the highest levels of inefficiency and time awake in bed compared to any other state. It is important to highlight that while these differences appear substantial within the context of the overall sample range, they translate to only modest absolute differences, with approximately 2% more time spent awake in the disturbed sleepers group. Despite the modest differences identified, previous literature has also identified poor sleep efficiency and increased wakefulness after sleep onset in patients with depression compared to controls [60].

A few sociodemographic differences were observed across the sleep subtypes, such as a higher prevalence of physical health comorbidities and a higher proportion of unemployment and sick leave in individuals with variable late sleep compared to the overall sample. These differences were only descriptive and not substantial enough to suggest clear, distinguishing features. The absence of such distinct differences indicates that the sleep subtypes may reflect more stable, inherent characteristics of the RADAR-MDD cohort rather than being heavily influenced by other participant characteristics. The finding that participants were most likely to stay in 1 subtype over time further suggests the presence of trait-like differences, indicating that these latent states may reflect underlying behavioral phenotypes rather than short-term fluctuations within the heterogeneous RADAR-MDD population. Nevertheless, transitions that did occur most commonly involved movement from more extreme profiles toward intermediate patterns (eg, high or low activity toward light or some activity; variable late sleepers toward efficient late sleepers), which may partly reflect regression to the mean as well as genuine behavioral change over time.

Depression severity differed across sleep states, although all states fell within the moderate range on the IDS-SR. State 4 (variable late sleepers) scored 10 points higher than state 1 (efficient early sleepers), representing a potentially clinically relevant difference in symptom severity. These findings are consistent with prior work, finding greater variability in sleep timings and later sleep-wake patterns associated with worse depression [11,22,49]. Together, these findings suggest that sleep subtypes may reflect differences in sleep-wake patterns and behavioral regularity within depression, ranging from stable and efficient sleep patterns to delayed and irregular patterns. By categorizing these relationships into possible subtypes, these findings provide an exploratory basis for a framework that identifies consistent sleep-depression profiles, which might mitigate the challenges presented by symptom heterogeneity in research and support more tailored circadian and sleep-focused approaches aimed at improving sleep-wake timing and regularity, such as structured sleep scheduling, sleep hygiene practices, or light exposure interventions.

#### Physical Activity Profiles Identified via RMT

In terms of physical activity measures, our subtyping analysis identified 3 distinct groups: a high-activity subtype with above-average levels of light, moderate, and vigorous activity, participating in nearly 1 hour of moderate-to-vigorous activity daily; a low-activity subtype with high levels of sedentary time (roughly 1.5 hours more than the high-activity group) and below-average levels across measures of physical activity with around 18 minutes spent in moderate-to-vigorous activity; and, finally, a group that presented a middle ground between these 2 extremes (light or some activity group), spending an average of 30 minutes daily in moderate-to-vigorous activity.

Previous research has identified various associations, including low gross physical activity and low moderate-to-vigorous physical activity with depression [14,61]. Research suggests the presence of a bidirectional link between low mood and physical inactivity, where symptoms of depression, such as feelings of anhedonia and low mood, can result in reduced motivation to engage in physical activity [62], when, in turn, low physical activity also increases the risk of depression [14]. Taken together, these findings suggest that the activity subtypes may reflect differing levels of behavioral activity and daily structure, ranging from highly engaged patterns to reduced activity consistent with behavioral withdrawal. Such patterns may be relevant for interventions aimed at increasing behavioral activation and daily activity. Employment status represents an important contextual factor, as individuals with depression might take leave from work, resulting in reduced activity levels. This study did not identify any meaningful descriptive differences in depression scores between the low, light, and high-activity subtypes, except for employment, where a slightly higher proportion of retired participants was found in the low-activity group compared to the overall sample.

### Strengths and Limitations

A strength of this study lies in its rigorous methodology for model identification. It emphasizes model robustness through grouped cross-validation to reduce the risk of overfitting the final model to the training data and enhancing generalizability [24]. It effectively addresses concerns about model instability by methodically managing the randomness associated with seed selection and reduces the risk of outlier-dependent performance by selecting the seed with the median LL value. Finally, applying this methodology to the dataset using 2 different clustering algorithms further improves the generalizability of the solution.

A core limitation of this study is the lack of external validation, which limits conclusions to the RADAR-MDD dataset. Future work should validate the model using (1) other depression cohorts and (2) through qualitative research with patients to examine the extent to which these data-derived groups reflect lived experience, work exceeding the scope of this analysis.

Another limitation of the methodological approach lies in the feature selection for the GMM and HMM models. Best practices in machine learning suggest starting with a broad set of features and using dimensionality reduction or feature selection techniques to retain the most influential variables that drive meaningful patterns in the data [24]. This paper could not use such techniques due to data availability and data quality; instead, it relied on previous literature and clinical relevance to justify feature inclusion. For example, measures of sleep stages were systematically excluded from the analysis due to insufficient evidence validating their accuracy [63,64]. As the validity of these measures improves, their inclusion in future subtyping work could lead to different or new profiles not identified here. Once achieved, future work could consider using a broader approach where all Fitbit features undergo data reduction before being included in the unsupervised learning models.

Another study limitation is the descriptive method used to outline clinical and demographic characteristics across the defined states. This method is limited as (1) each data point was placed in its most probable cluster without considering the likelihood of belonging to multiple clusters; (2) comparisons were made among observations instead of participants, which may distort the findings in favor of individuals with more repeated observations; and (3) the descriptive nature of this approach hinders our ability to assess the predictive capability of these subtypes in assessing depression scores. In addition, although missing data were minimal, k-nearest neighbors’ imputation was used to preserve longitudinal trajectories, which may introduce minor smoothing of the data, although any impact is likely minimal.

We acknowledge the vast heterogeneity observed in the RADAR-MDD dataset. Participants were not necessarily in an acute episode of depression throughout the 2-year follow-up, resulting in a diverse set of observations with varying levels of severity and caseness in the sample. While this can be considered an advantage since it reflects the real-world complexity of depression, it also restricts the ability to confidently determine whether the differences observed are traits found in the general population or unique to individuals with an acute episode.

### Conclusions

In conclusion, previous research suggests that various digital markers linked to sleep and activity can serve as predictors of depression. Therefore, we posited that these digital indicators could highlight phenotypic differences in patients and assist in identifying meaningful subtypes in the RADAR-MDD population. The identified subtypes correspond well to previously established links between depression and both objective and subjective measures of sleep and activity. By categorizing these relationships into potential subtypes, the findings lay the groundwork for a framework that identifies consistent depression profiles, potentially alleviating the challenges posed by symptom diversity in research and clinical practice. While further validation is required, such profiles may support more personalized patient management by informing targeted approaches to sleep timing and regularity, promoting daily activity and engagement, and enabling more context-aware monitoring. By summarizing complex behavioral patterns into clinically meaningful profiles, this approach may also facilitate early recognition of meaningful behavioral changes over time.

## Supplementary material

10.2196/86900 Multimedia Appendix 1 Tables and figures with further information relevant to the paper.

## Abbreviations

*DSM-5*: *Fifth Edition of the Diagnostic and Statistical Manual*
GAD-7: 7-item Generalized Anxiety Disorder
GMM: Gaussian mixture model
HMM: hidden Markov model
*ICD-11*: *Eleventh Revision of the International Classification of Diseases*
IDS-SR: Inventory of Depressive Symptomatology–Self Report
LL: log-likelihood
MDD: major depressive disorder
RADAR-MDD: Remote Assessment of Disease and Relapse Study in Major Depressive Disorder study
RMT: remote measurement technology
WSAS: Work and Social Adjustment Scale
