# Supplementary material for: Sleep and Activity Patterns in Depression From Wearable Data: Unsupervised Clustering Study
Source: J Med Internet Res. 2026 Jun 10;28:e86900. doi: 10.2196/86900 (PMC13251678; doi:10.2196/86900)

Multimedia Appendix

Table S1. Descriptive means for the sleep features included in the four-state HMM solution in scaled and raw units.

Table S2. Descriptive means for the physical activity features included in the three-state HMM solution in scaled and raw units.

Table S3. Descriptive means for the sleep features included in the four-cluster GMM and four-state HMM solutions.

Table S4. Descriptive means for the physical activity features included in the seven-cluster GMM and seven-state HMM solutions.

Table S5. Descriptive means for the physical activity features included in the three-cluster GMM and three-state HMM.

Figure S1. The mean log-likelihood score versus the number of clusters in GMM and HMM sleep feature models.

Figure S2. The mean log-likelihood versus the number of clusters/states in GMM (Gaussian mixture model) and HMM (Hidden Markov model) physical activity feature models.

Table S1. Descriptive means for the sleep features included in the four- state HMM solution in scaled and raw units.

	**Units**	**Efficient Early**<br>Obvs. = 565	**Efficient Late**<br>Obvs. = 731	**Disrupted**<br>Obvs. = 463	**Variable Late**<br>Obvs. = 271
Total Sleep Time (M)	Scaled	0.19	-0.13	-0.03	0.00
	total duration (hours) *	7 hrs 43 min	7 hrs 19 min	7 hrs 26 min	7 hrs 29 min
Sleep Onset (M)	Scaled	-0.38	0.33	-0.45	0.68
	Local time *	23:40	00:47	23:32	01:22
Sleep Offset (M)	Scaled	-0.28	0.17	-0.43	0.85
	Local time *	07:39	08:25	07:24	09:33
Sleep Efficiency (M)	Scaled	0.40	0.22	-0.61	-0.40
	% of total sleep time to time in bed	92.38	92.00	90.18	90.64
Time Spent Awake (M)	Scaled	-0.40	-0.22	0.61	0.40
	% of total time in bed to time awake	7.62	8.00	9.82	9.36
Total Sleep Time (SD)	Scaled	-0.39	0.15	-0.24	0.80
	7-day variation (in hours) *	1 hr 1 min	1 hr 29 min	1 hr 8 min	2 hr 3 min
Sleep Onset (SD)	Scaled	-0.41	0.11	-0.22	0.92
	7- day variation (in hours) *	47 min	1 hr 18 min	58 min	2 hrs 7 min
Sleep Offset (SD)	Scaled	-0.31	0.13	-0.32	0.84
	7- day variation (in hours) *	58 min	1 hr 25 min	57 min	2 hrs 7 min
Sleep Efficiency (SD)	Scaled	-0.16	-0.04	0.10	0.29
	7- day variation (in %) *	2.73	2.86	3.03	3.25

Note. *Raw scores were converted into standard time format or hours (hrs) and minutes (min) for readability. Obvs = number of observations. Scaled = deviations from the population mean.

Table S2. Descriptive means for the physical activity features included in the three- state HMM solution in scaled and raw units.

	Units	High Activity<br>582	Light/Some Activity<br>709	Low Activity<br>457
**Sedentary Time (M)**	Scaled	-0.29	0.12	0.18
	In hours *	18 hrs 26 min	19 hrs 34 min	19 hrs 44 min
**Light Activity (M)**	Scaled	0.22	0.02	-0.31
	In hours *	3 hrs 16 min	2 hrs 57 min	2 hrs 26 min
**Moderate Activity (M)**	Scaled	0.40	-0.10	-0.35
	In hours *	26 min	15 min	10 min
**Vigorous Activity (M)**	Scaled	0.42	-0.07	-0.43
	In hours *	26 min	15 min	8 min
**Nighttime Activity (M)**	Scaled	0.42	-0.23	-0.18
	In hours *	22 min	9 min	10 min
**Total Calories Burned (M)**	Scaled	0.24	0.00	-0.30
	Total calories burned	2253	2103	1914
**Sedentary Time (SD)**	Scaled	0.15	-0.15	0.04
	7-day variation (in hours) *	2 hrs 13 min	1 hrs 42 min	2 hrs 2 min
**Light Activity (SD)**	Scaled	0.23	-0.09	-0.16
	7-day variation (in hours) *	1 hrs 9 min	57 min	55 min
**Moderate Activity (SD)**	Scaled	0.37	-0.08	-0.36
	7-day variation (in hours) *	21 min	14 min	10 min
**Vigorous Activity (SD)**	Scaled	0.38	-0.03	-0.44
	7-day variation (in hours) *	21 min	14 min	8 min
**Nighttime Activity (SD)**	Scaled	0.39	-0.22	-0.16
	7-day variation (in hours) *	17 min	8 min	9 min
**Total Calories Burned (SD)**	Scaled	0.35	-0.15	-0.22
	7- day variation (total calories burned) *	413	298	283

Note. *Raw scores were converted into standard time format or hours (hrs) and minutes (min) for readability. Obvs = number of observations. Scaled = deviations from the population mean.

Table S4. Descriptive means for the physical activity features included in the seven- cluster GMM and seven- state HMM solutions.

	State/Cluster	# Obvs.	ST (M)	Li. A (M)	Mo.A (M)	Vi. A (M)	NT. A (M)	TDC (M)
GMM	1	170	-0.71	0.77	1.81	1.40	1.38	1.07
	2	187	-1.01	-0.45	-0.26	-0.24	0.03	-0.66
	3	206	0.12	-0.15	-0.63	-0.70	-0.18	-0.45
	4	232	0.26	-1.40	-0.82	-0.78	-0.52	-1.07
	5	302	0.08	0.62	0.07	0.11	0.69	0.37
	6	321	0.09	0.33	0.47	0.64	-0.44	0.55
	7	330	0.52	0.05	-0.33	-0.33	-0.46	-0.02
HMM	1	125	-0.54	0.25	1.15	0.99	0.86	0.66
	2	138	-0.15	-0.33	-0.34	-0.42	-0.18	-0.47
	3	148	0.57	-0.51	-0.37	-0.44	-0.22	-0.17
	4	237	-0.20	0.13	-0.05	-0.11	0.06	-0.08
	5	281	-0.21	0.24	0.11	0.15	0.51	0.04
	6	336	-0.01	0.08	0.21	0.42	-0.27	0.23
	7	483	0.23	-0.07	-0.28	-0.33	-0.25	-0.13

Continued on the next page.

	State/Cluster	ST (SD)	Li. A (S)	Mo. A (SD)	Vi. A (SD)	NT.A (SD)	TDS (SD)
GMM	1	0.35	0.56	1.67	1.39	1.34	1.20
	2	1.35	0.81	-0.06	0.01	0.09	1.20
	3	0.14	0.42	-0.56	-0.74	-0.21	-0.17
	4	0.38	-1.04	-0.95	-0.94	-0.53	-0.55
	5	-0.43	0.04	0.04	0.17	0.74	-0.27
	6	-0.27	0.14	0.45	0.52	-0.44	0.09
	7	-0.65	-0.45	-0.28	-0.27	-0.49	-0.65
HMM	1	0.32	0.29	1.05	0.87	0.73	0.81
	2	0.46	-0.07	-0.40	-0.46	-0.15	-0.01
	3	-0.28	-0.38	-0.35	-0.49	-0.24	-0.40
	4	0.19	0.19	-0.02	-0.08	0.12	0.14
	5	0.07	0.18	0.07	0.17	0.47	0.09
	6	-0.07	-0.01	0.22	0.36	-0.22	0.13

State/Cluster	ST (SD)	Li. A (S)	Mo. A (SD)	Vi. A (SD)	NT.A (SD)	TDS (SD)
7	-0.21	-0.13	-0.23	-0.25	-0.25	-0.30

Note. ST= Sedentary Time; Li.A= Light Activity; Mo.A= Moderate Activity; Vi.A= Vigorous Activity; NT.A= Nighttime Activity; TDC= Total Daily Calories Burned. HMM = Hidden Markov Model; GMM = Gaussian Mixture Model. Obvs. = Number of observations in the state/cluster.

Table S5. Descriptive means for the physical activity features included in the three- cluster GMM and three- state HMM.

	Gaussian Mixture Model			Hidden Markov Model		
**Cluster/ State**	**Cluster 1**	**Cluster 2**	**Cluster 3**	**State 1**	**State 2**	**State 3**
# Obvs.	671	845	232	582	709	457
Sedentary Time (M)	-0.60	0.40	0.26	-0.29	0.12	0.18
Light Activity (M)	0.27	0.17	-1.40	0.22	0.02	-0.31
Moderate Activity (M)	0.52	-0.19	-0.82	0.40	-0.10	-0.35
Vigorous Activity (M)	0.47	-0.16	-0.78	0.42	-0.07	-0.43
Nighttime Activity (M)	0.52	-0.27	-0.52	0.42	-0.23	-0.18
Total Calories (M)	0.23	0.11	-1.07	0.24	0.00	-0.30
Sedentary Time (SD)	0.49	-0.50	0.38	0.15	-0.15	0.04
Light Activity (SD)	0.49	-0.11	-1.04	0.23	-0.09	-0.16
Moderate Activity (SD)	0.54	-0.17	-0.95	0.37	-0.08	-0.36
Vigorous Activity (SD)	0.50	-0.14	-0.94	0.38	-0.03	-0.44
Nighttime Activity (SD)	0.54	-0.28	-0.53	0.39	-0.22	-0.16
Total Calories (SD)	0.73	-0.42	-0.55	0.35	-0.15	-0.22

Note. Obvs. = Number of observations in the state/cluster.

Table S3. Descriptive means for the sleep features included in the four- cluster GMM and four-state HMM solutions.

	Gaussian Mixture Model				Hidden Markov Model			
**Cluster/State**	**C1**	**C2**	**C3**	**C4**	**S 1**	**S 2**	**S 3**	**S 4**
# of Obs.	669	618	495	248	565	731	463	271
Total Sleep Time (M)	0.22	-0.17	-0.19	0.21	0.19	-0.13	-0.03	0.00
Sleep Onset Time (M)	-0.42	0.35	-0.26	0.77	-0.38	0.33	-0.45	0.68
Sleep Offset Time (M)	-0.31	0.11	-0.31	1.18	-0.28	0.17	-0.43	0.85
Sleep Efficiency (M)	0.50	0.53	-1.04	-0.60	0.40	0.22	-0.61	-0.40
Time Awake (M)	-0.50	-0.53	1.04	0.60	-0.40	-0.22	0.61	0.40
Total Sleep Time (SD)	-0.64	0.30	-0.25	1.47	-0.39	0.15	-0.24	0.80
Sleep Efficiency (SD)	-0.33	-0.09	0.31	0.51	-0.16	-0.04	0.10	0.29
Sleep Onset Time (SD)	-0.56	0.16	-0.18	1.47	-0.41	0.11	-0.22	0.92
Sleep Offset Time (SD)	-0.55	0.22	-0.27	1.44	-0.31	0.13	-0.32	0.84

Figure S1. The mean log-likelihood score versus the number of clusters in GMM and HMM sleep feature models.

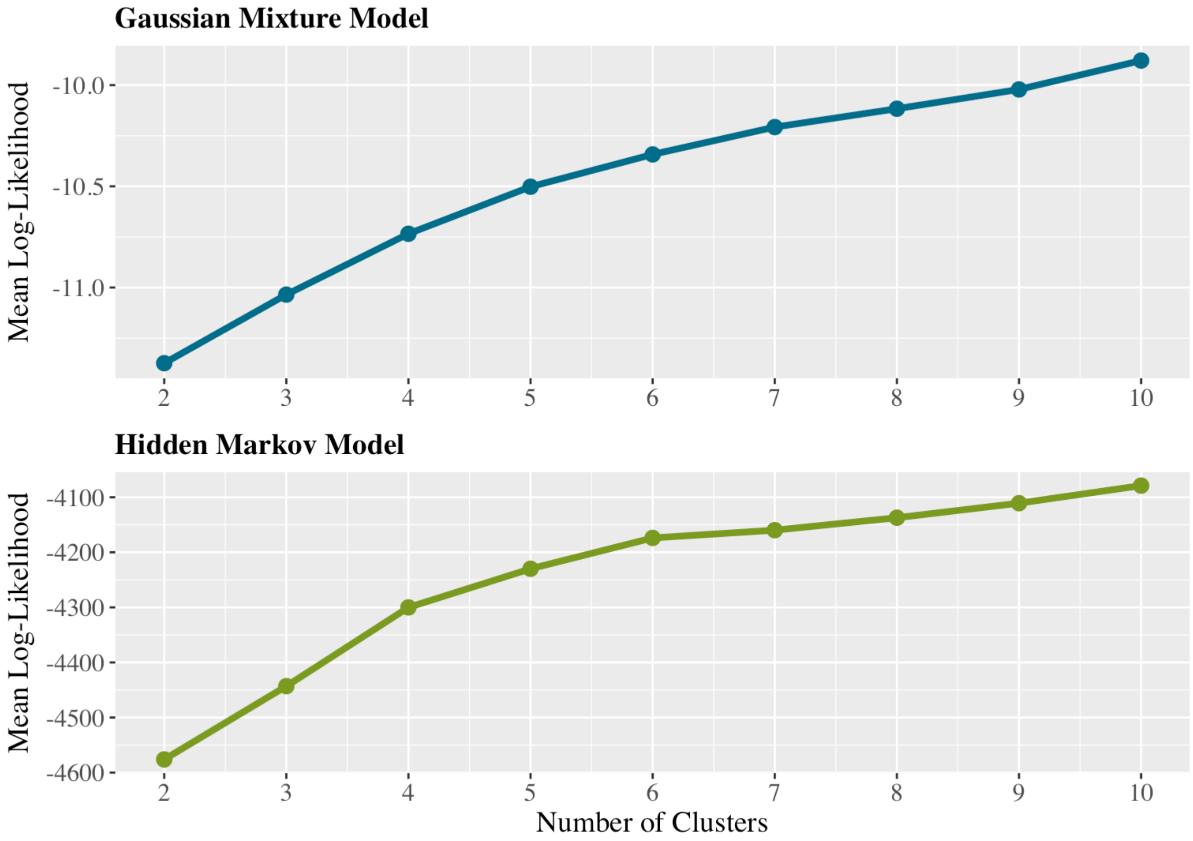

Figure S2. The mean log-likelihood versus the number of clusters/states in GMM (Gaussian mixture model) and HMM (Hidden Markov model) physical activity feature models.

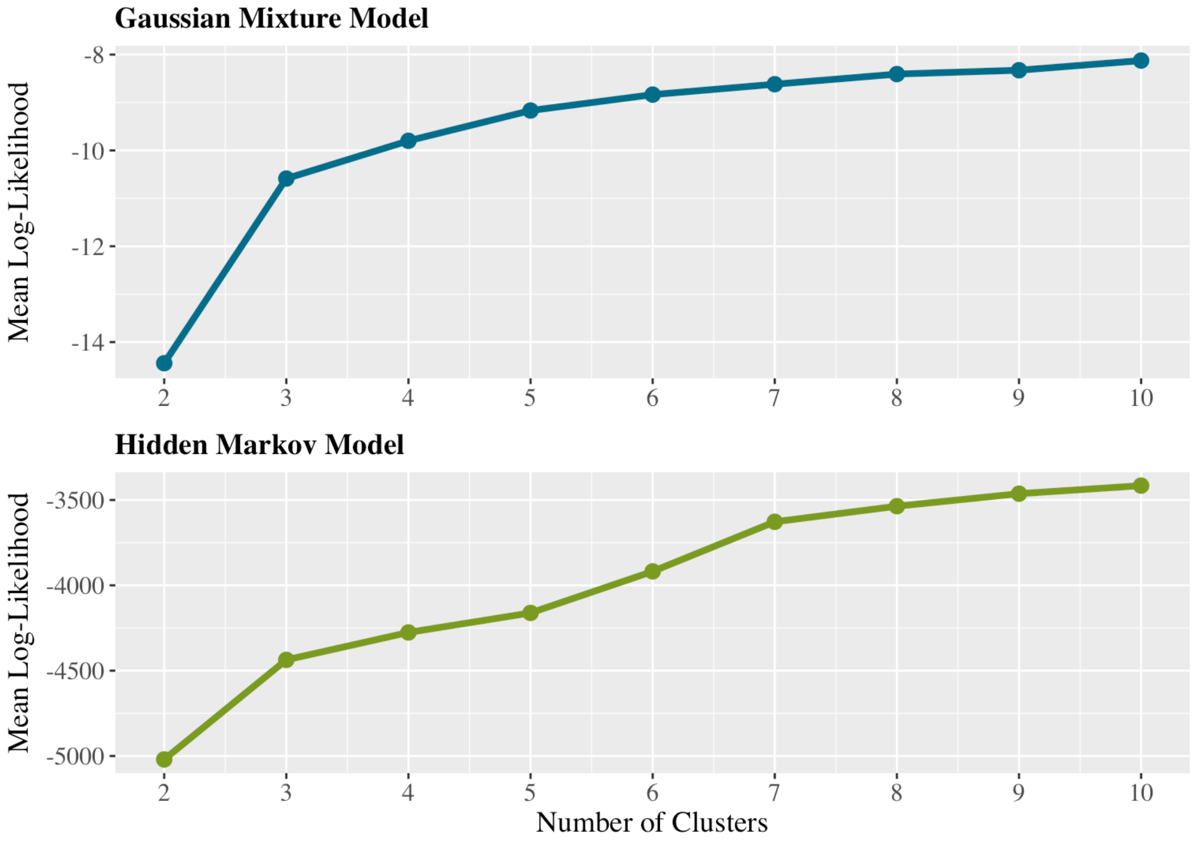

Supplement: Multimedia Appendix 1 [file jmir-v28-e86900-s001.pdf]
